# Fetal Alcohol Exposure and Attention: Moving Beyond ADHD

**Published:** 2001

**Authors:** Claire D. Coles

**Affiliations:** Claire D. Coles, Ph.D., is a professor in the Departments of Psychiatry and Behavioral Sciences and Pediatrics at Emory University School of Medicine and is director of the Fetal Alcohol Center at the Marcus Institute, a division of the Kennedy-Krieger Institute at Emory University, Atlanta, Georgia

Clinical descriptions of children with fetal alcohol syndrome (FAS) and other alcohol-related disabilities^1^ often cite attention deficit hyperactivity disorder (ADHD) (American Psychiatric Association [APA] 1994) as a central feature of the behavioral characteristics associated with prenatal exposure to alcohol (Oesterheld and Wilson 1997). The assumption that prenatal alcohol exposure produces ADHD and other attention problems is based on case study reports and descriptions of patients identified through clinical practice (Steinhausen et al. 1993). The association between prenatal alcohol exposure and ADHD is also supported by results from prospective studies of alcohol-exposed children who do not have FAS (e.g., Streissguth et al. 1995). However, not all empirical studies of alcohol-exposed children have found either the behaviors characteristic of ADHD or deficits on measures of attention on neurocognitive tests (e.g., Boyd et al. 1991; Brown et al. 1991; Fried et al. 1992). These discrepancies, in addition to the debate about the ADHD diagnosis itself (see Barkley 1998; Shaywitz et al. 1994), raise a number of questions about the association between ADHD and prenatal alcohol exposure.

The clinical diagnosis of ADHD, although usually reliable, is not based on neurocognitive tests of attention but relies, instead, on clinical observation and on parent and teacher reports. The behaviors measured, therefore, reflect deficits in attention only by inference. In contrast, the methods of neuropsychology and cognitive development use experimental procedures to examine behaviors that reflect the fundamental processes of attention (Mirsky et al. 1991).

To examine the effect of prenatal alcohol exposure on attention factors, as well as the relationship between these factors and the ADHD diagnosis, we (Coles et al. 1997) compared ADHD school-aged children with children diagnosed with either FAS or partial FAS. For this research, we used two sets of criteria.

The first set of criteria was the “traditional” clinical method for diagnosing ADHD using observations of behavior; parent and teacher checklists (e.g., the Child Behavior Checklist [CBCL] [Achenbach 1991] and SNAP [Swanson et al. 1982]); and cognitive tasks typically used by clinicians (e.g., the Wechsler Intelligence Scale for Children, Revised [WISC–R] and Digit Span [Wechsler 1991]). See table 1 for descriptions of these tests.

The second set of criteria was the neuropsychological model developed by Mirsky and colleagues (1991), one of several competing models of attention based on neuropsychological studies. At the time of our study, this model proposed four factors of attention that could be measured using neuropsychological tests. (Another factor has since been added.) The four factors we used were focus, shift, sustain, and encode. *Focus* refers to the ability to attend selectively to appropriate information. *Shift* refers to the ability to allocate attentional resources—that is, the ability to shift attention from one task to another when appropriate. *Sustain* refers to the ability to maintain a focused alertness in perceiving a signal. *Encode* refers to the ability to maintain information in the working memory while performing some cognitive process using that information (e.g., by manipulating or memorizing symbols). In contrast to the ADHD diagnosis, understanding attention in this manner facilitates the study of both the characteristics of attention itself and the differential effects of prenatal alcohol exposure on these more specific factors.

In our (Coles et al. 1997) study, we measured the focus dimension using the WISC–R Coding subtest (Wechsler 1991); the shift dimension with the Wisconsin Card Sorting Test (WCST) (Grant and Berg 1980); the sustain dimension with a computerized vigilance procedure known as the Continuous Performance Test (CPT); and the encode dimension with the Number Recall and Arithmetic subtests from the Kaufman Assessment Battery for Children (K–ABC) (Kaufman and Kaufman 1987). In addition, we used a paired associated task called the Zoo Task (Conte et al. 1986). See table 2 for descriptions of these tests.

It is necessary to control for the social factors, such as social class and caregiving resources, that can be confounded with prenatal exposure effects when clinical samples of children diagnosed with FAS are compared with community samples. For this reason, the study sample was recruited from a longitudinal cohort of 149 children (with an average age of 7.63 years)—who were of low socioeconomic status (SES) and predominantly African-American—and their caregivers. The sample included 25 alcohol-exposed children who were physically affected (i.e., had either FAS or fetal alcohol effects [FAE]); 62 alcohol-exposed children who were not affected; and a control group, consisting of 35 children who had not been exposed to alcohol during pregnancy but who were selected from the same low-SES population. We recruited the study participants when mothers came to a hospital clinic for prenatal care. We documented maternal alcohol use at that time and examined the infants at birth. We also documented the physical effects of alcohol exposure in the newborn period and at followup using a Dysmorphia Checklist. To examine the relationship with ADHD, we selected 27 ADHD-diagnosed children from the child psychiatry clinic at the same hospital where the other children were born. We then matched the ADHD children to the children in our study according to age, SES, and ethnic identification. Maternal alcohol use during and after pregnancy was based on maternal self-report, hospital records, and laboratory tests.

The FAS–FAE and ADHD groups had similar scores on intelligence tests and both were lower than those in the other two groups (i.e., the control group and the alcohol-exposed but not affected group). When the conventional ADHD diagnostic model was used, children with ADHD were identified reliably with standard checklists (e.g., CBCL and SNAP), which were completed by parents and teachers, as well as the teacher version of the Diagnostic Interview Schedule for Children (DISC–T) (Costello et al. 1984). Findings indicated that these measures were effective in identifying children with an ADHD diagnosis. In contrast, the FAS–FAE children’s scores on these measures were equivalent to those of the control group. Thus, for this group of alcohol-affected children, prenatal alcohol exposure was not associated with ADHD as it is usually diagnosed.

We also evaluated the four attention-related factors. Coding (i.e., focus), efficiency on the WCST (i.e., shift), reaction time and error rate on the computerized sustained-attention tasks (i.e., sustain), and paired associate learning (i.e., encode) differed among our groups of 7-year-olds. However, the performance pattern was not the same for children with ADHD as those with FAS–FAE (see figure). As theory would predict, the children with ADHD performed least well on measures of focused and sustained attention. In contrast, children in the FAS–FAE group performed least well on measures of encoding and shifting attention. These results suggested that these two groups of children had unique attentional profiles. Those in the ADHD group had difficulty in selective and sustained attention and were more impulsive. Based on parents’ and teachers’ reports, the ADHD children had more behavioral problems. Conversely, the children in the FAS–FAE group were not impulsive and were not identified as having significant behavior problems. However, these children had more difficulty in learning new material (i.e., encode) and in utilizing flexibility in problem-solving (i.e., shift). In addition, on other tests given at the same time, they had problems completing tasks requiring visuo-spatial processing (e.g., copying a model figure) and in performing some academic processes (e.g., math).

These results call into question the assumption that behavior seen in children with FAS results from the same neurocognitive deficits as those seen in children diagnosed with ADHD. Our comparison of these groups of children showed that even though their impairment on tests of global intelligence was similar, little similarity existed in their pattern of responses. Furthermore, their behavior problems also differed.

More research on these issues is necessary. Although similar to some findings (Boyd et al. 1991; Fried et al. 1992), these results are inconsistent with reports that have identified deficits in focusing and sustaining attention in alcohol-exposed longitudinal samples (e.g., Kopera-Frye et al. 1997; Streissguth et al. 1995) and ADHDlike behavior among FAS-FAE patients (Nanson and Hiscock 1990). To understand how prenatal alcohol exposure affects development, it is probably insufficient to examine only its effect on social behavior and emotions (i.e., items examined in DSM–IV [APA 1994]). Instead, researchers should evaluate development progression in terms of both the many factors that affect it and the many processes that comprise behavior. For example, when compared with clinically referred children, the examination of children diagnosed with FAS-FAE who have been followed longitudinally may lead to different results, because their life experiences differ. For the most part, children in our study (Coles et al. 1997) had not experienced the highly disorganized and abusive caregiving situations that are often associated with clinical referral and which may affect outcomes in studies that use clinical samples. These outcomes suggest that understanding the relationship between prenatal alcohol exposure and attention will require a multifaceted approach that moves beyond characterizing behavioral deficits as ADHD. Drawing on the methods of developmental psychology to examine cognitive and behavioral processes and the many factors that influence them will provide insight into both the effects of prenatal alcohol exposure and the nature of development itself. By identifying specific cognitive processes affected by alcohol and documenting their effect on academic and behavioral functioning, practical teaching methods can be developed to support the education of affected children and prevent the development of secondary disabilities.

## Figures and Tables

**Figure:**
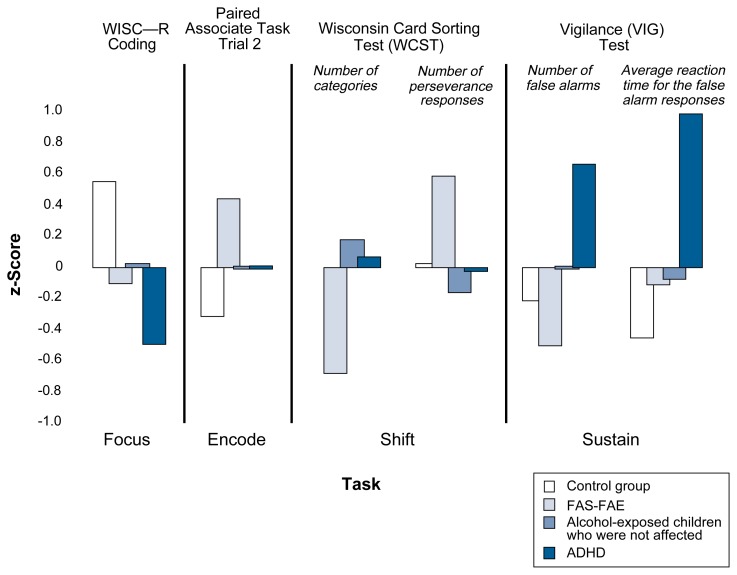
Performance on attention tasks by FAS-FAE and ADHD status. ADHD = attention deficit hyperactivity disorder; FAE = fetal alcohol effect; FAS = fetal alcohol syndrome; WISC-R = Wechsler Intelligence Scale for Children, Revised. NOTE: The bars represent the variation away from the average score (i.e., the z-score) so that the bigger the bar, the more the group differs from “normal.”

**Table 1:** Tests Used To Diagnose ADHD and Cognition

Behavior	Definition	Test Used	Test Description
ADHD-related behavior problems	Behaviors characteristic of ADHD, including restlessness, impulsivity, distractibility, inattention	Child Behavior Checklist (CBCL)	Checklist includes many problem behaviors of school-age children and is completed by a parent or teacher
		SNAP	Checklist includes a number of ADHD-related behaviors and is completed by a parent
		Diagnostic Interview Schedule for Children, Teacher Version (DISC–T)	Structured interview administered to a child’s teacher to permit DSM–IV diagnosis
Intelligence	General ability to participate in school activities	Kaufmans Assessment Battery for Children (K–ABC); Wechsler Intelligence Scale for Children, Revised (WISC–R)	Individually administered tests of aptitude (usually called IQ tests) given to a child in a standardized manner by a psychologist
Auditory processing of information	Ability to attend to and understand sounds	Digit Span	As part of the IQ battery, a child is read a series of numbers and then has to repeat them

ADHD = attention deficit hyperactivity disorder; Digit Span is a component of the WISC-R; DSM–IV = the *Diagnostic and Statistical Manual of Mental Disorders, Fourth Edition,* published by the American Psychiatric Association.

**Table 2:** Tests Assessing Cognitive Aspects of Attention

Dimension of Attention	Definition	Test Used	Task Description
Focus	Selective attention to appropriate stimuli	WISC–R Coding	The child must rapidly identify and write in symbols associated with numbers
Shift	Appropriate flexibility in response to new information; allocation of attentional resources	Wisconsin Card Sorting Test (WCST)	The child must sort cards based on one of three underlying principles: color, shape, or number of items on card. When the sorting category is guessed, it is changed. Few categories and perseverance on the wrong category indicate lack of flexibility
Sustain	Ability to maintain alert state and attention to task	Continuous Performance Test (CPT) (also called Vigilance [VIG] Test)	From letters rapidly displayed on a computer screen, the child must identify a predesignated signal without missing letters or responding impulsively to wrong letters (i.e., false alarms). Reaction time also is measured
Encode	Ability to learn new material and manipulate material in working memory while processing into long-term memory	• Paired Associate (PA) Task (also called Zoo Task)	Cards with animals are repeatedly paired with “zoo homes” of different colors. The child must recall the correct zoo when presented with the animal card
		• Number Recall subtest from the Kaufman Assessment Battery for Children (K–ABC)	The child is read a series of numbers and must repeat them accurately
		• Arithmetic subtest from the K–ABC	The child must display basic math skills

WISC–R = Wechsler Intelligence Scale for Children, Revised.

## Footnotes

1 See article on pp. 153–158 for definitions of FAS and other related conditions.
